# Identification of a PRPF4 Loss-of-Function Variant That Abrogates U4/U6.U5 Tri-snRNP Integration and Is Associated with Retinitis Pigmentosa

**DOI:** 10.1371/journal.pone.0111754

**Published:** 2014-11-10

**Authors:** Bastian Linder, Anja Hirmer, Andreas Gal, Klaus Rüther, Hanno Jörn Bolz, Christoph Winkler, Bernhard Laggerbauer, Utz Fischer

**Affiliations:** 1 Department of Biochemistry, University of Würzburg, Würzburg, Germany; 2 Department of Human Genetics, University Medical Center Hamburg-Eppendorf, Hamburg, Germany; 3 Department of Ophthalmology, Sankt Gertrauden-Krankenhaus, Berlin, Germany; 4 Institute of Human Genetics, University Hospital of Cologne, Cologne, Germany; 5 Bioscientia Center for Human Genetics, Ingelheim, Germany; 6 Department of Biological Sciences, National University of Singapore, Singapore, Singapore; International Centre for Genetic Engineering and Biotechnology, Italy

## Abstract

Pre-mRNA splicing by the spliceosome is an essential step in the maturation of nearly all human mRNAs. Mutations in six spliceosomal proteins, PRPF3, PRPF4, PRPF6, PRPF8, PRPF31 and SNRNP200, cause retinitis pigmentosa (RP), a disease characterized by progressive photoreceptor degeneration. All splicing factors linked to RP are constituents of the U4/U6.U5 tri-snRNP subunit of the spliceosome, suggesting that the compromised function of this particle may lead to RP. Here, we report the identification of the p.R192H variant of the tri-snRNP factor PRPF4 in a patient with RP. The mutation affects a highly conserved arginine residue that is crucial for PRPF4 function. Introduction of a corresponding mutation into the zebrafish homolog of PRPF4 resulted in a complete loss of function *in vivo*. A series of biochemical experiments suggested that p.R192H disrupts the binding interface between PRPF4 and its interactor PRPF3. This interferes with the ability of PRPF4 to integrate into the tri-snRNP, as shown in a human cell line and in zebrafish embryos. These data suggest that the p.R192H variant of PRPF4 represents a functional null allele. The resulting haploinsufficiency of PRPF4 compromises the function of the tri-snRNP, reinforcing the notion that this spliceosomal particle is of crucial importance in the physiology of the retina.

## Introduction

Mutations that interfere with splicing are a frequent cause of human disease [1], [2]. The majority of these mutations affect pre-mRNA splice sites or regulatory elements in *cis* [3]. However, also mutations that interfere with the trans-acting machinery that catalyzes the splicing reaction – the spliceosome – can lead to disease [4]–[7]. Surprisingly, despite the essential role of pre-mRNA splicing for gene expression on a transcriptome-wide scale, mutations in general splicing factors are often associated with specific phenotypes that are restricted to a particular tissue or even cell type [2]. A prime example for such a tissue specificity paradox is the hereditary eye disease retinitis pigmentosa (RP), which is characterized by a selective loss of photoreceptor cells in the retina. As a consequence, patients suffer from a severe visual handicap [8]. Although this phenotype is highly selective and affects primarily rod photoreceptors, a substantial fraction of RP- cases have been linked to mutations in the general splicing factors PRPF3, PRPF4, PRPF6, PRPF8, PRPF31 and SNRNP200 [9]–[14].

The aforementioned proteins are part of the spliceosome, a highly dynamic molecular machine that consists of five small nuclear ribonucleoprotein particles (snRNPs; termed according to their RNA content U1, U2, U4/U6 and U5) and a large set of non-snRNP factors [15]. It is formed in a stepwise manner on the *cis*-acting sequence elements of introns, including the branch point and the 5′ and 3′ splice sites, which define the boundaries to exons. In a first step, U1 and U2 snRNPs bind to the 5′-splice site and the branch point, respectively. The resulting pre-spliceosomal complex then recruits U4/U6 and U5 snRNPs, which form prior to their integration into the spliceosome the so-called U4/U6.U5-tri-snRNP. Structural rearrangements then lead to the release of U1 and U4 snRNPs, and the formation of the catalytically active spliceosome. After splicing, the spliceosome is disassembled and its individual subunits are recycled so that they can re-assemble on another intron. While U1 and U2 snRNPs are recycled as “mono-snRNPs”, the tri-snRNP is rebuilt by the joining of the snRNPs U4 and U6, which generates a U4/U6 di-snRNP that is subsequently bound by the U5 snRNP [16].

Intriguingly, every splicing factor linked to RP thus far is a component of the U4/U6.U5 tri-snRNP. The tri-snRNP is essential for the formation of the active center of the spliceosome, participates in splice site selection and has been recently proposed to recruit the nuclear RNA surveillance machinery to mRNAs [17]–[19]. It has been hypothesized that defects in RP-linked splicing factors affect gene expression in a manner to which the retina is uniquely sensitive [20], [21]. In line with this hypothesis, we have recently shown in a zebrafish model for RP that the deficiency of PRPF31 predominantly affects the expression of retina-specific transcripts [22]. Many of the affected transcripts were also associated with retinal disease, thereby providing a direct link between defects in general splicing factors and a retinal phenotype. Of note, the knockdown of another tri-snRNP protein, PRPF4, resulted in a photoreceptor-specific phenotype that was indistinguishable from that of the PRPF31 model [22]. Therefore, we reasoned that PRPF4 could be similarly involved in the pathogenesis of RP and screened patients for mutations in the *PRPF4* gene.

Here, we characterize the molecular and physiological effects of a missense mutation (p.R192H) in the tri-snRNP factor, *PRPF4*, which was identified in a sporadic RP patient. Several lines of evidence suggest that this variant represents a loss-of-function allele. First, a corresponding mutation in the zebrafish PRPF4 homolog rendered it physiologically inactive. Second, binding of PRPF4 to its interactor PRPF3 was abrogated by the p.R192H amino acid exchange. Third, variant PRPF4 failed to integrate into the tri-snRNP in human cell lines and zebrafish embryos. Together, these findings indicate that PRPF4 haploinsufficiency may contribute to RP. Furthermore, they underline the role of tri-snRNP splicing factors as mutational hotspots in RP and demonstrate the feasibility of a direct analysis of these candidate genes in RP patients.

## Results

### Identification of a loss-of-function variant of the splicing factor PRPF4 in a patient with RP

**Figure 1:**
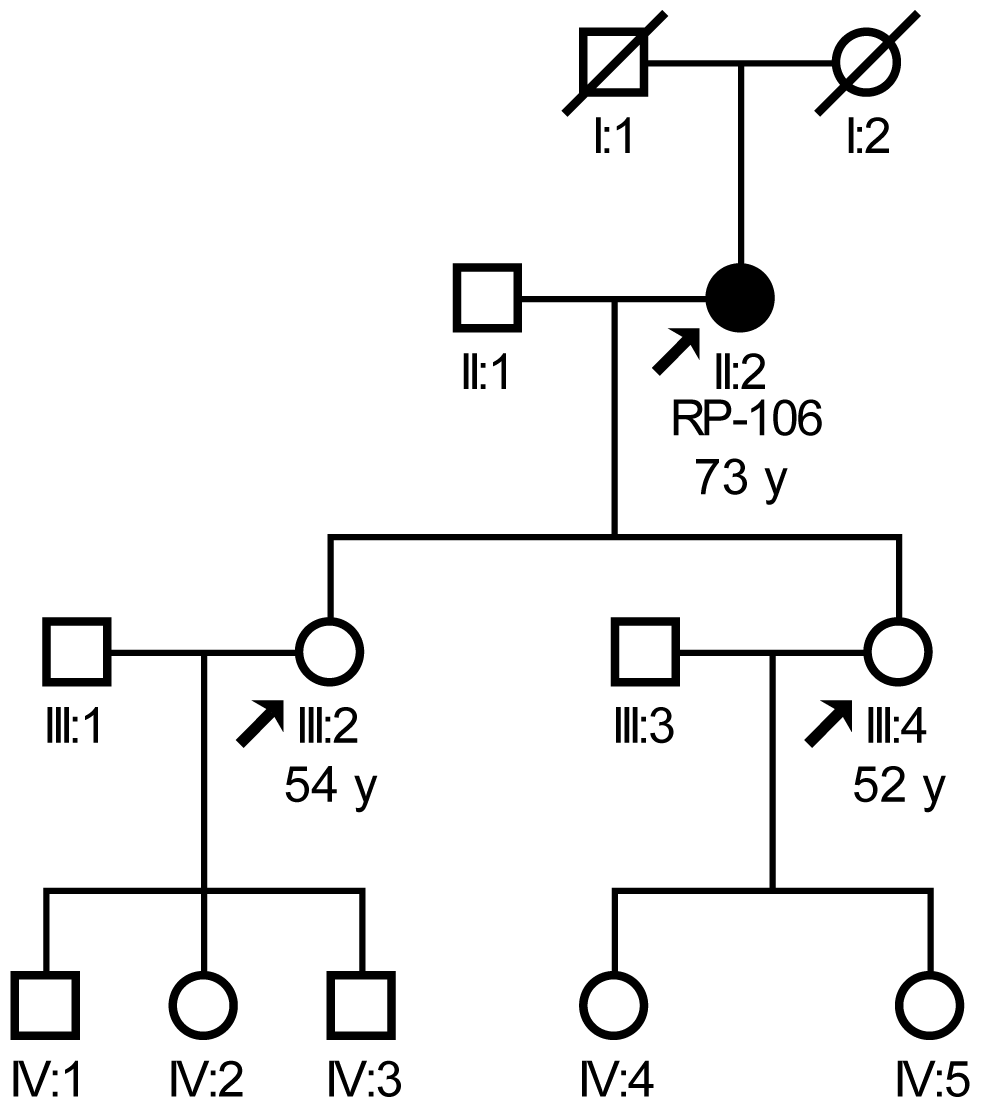
RP family described herein. II:2, index patient and heterozygous carrier of the PRPF4 variant p.R192H. Arrows, individuals who were available for genetic and clinical characterization. Circles, female individuals; Squares, male individuals. Filled symbols indicate affected persons. White symbols, unaffected persons by detailed ophthalmological investigation (III:2, III:4) or medical history. III:4, healthy carrier of the PRPF4 variant. Crossed symbols, deceased persons. Age at the time of examination is given below the pedigree symbols.

We screened DNA samples from 85 patients with sporadic or autosomal dominant RP (adRP) for mutations in *PRPF4*. This revealed a heterozygous non-synonymous nucleotide exchange, c.575G>A, in the sporadic female patient RP-106 (Fig. 1A). At the age of 39 years, RP-106 had stopped driving because of night blindness. Constriction of the visual field was noted at the age of approximately 45 years. With 55 years of age, early cataracts were found. Funduscopy showed pale papillae, narrowing of retinal vessels, and bone spicule-like pigmentation in the periphery. The scotopic electroretinogram (ERG) was extinguished, the photopic ERG was almost extinguished. RP-106 has two daughters (III:2, 54 years, and III:4, 52 years). III:4 was found to carry the c.575G>A variant. III:2 and III:4 underwent detailed ophthalmological investigation including assessment of visual acuity, funduscopy, perimetry, dark adaptation and ERG and did not show any signs of retinal degeneration. As shown in a recent study by Chen et al. [10], the onset of PRPF4-associated RP is variable (between 15 and 27 years of age). Both inter- and intrafamilial clinical variability are common in RP. The reported onset of visual complaints (20 years of age) in our patient corresponds to the range reported by Chen et al [10].

Although c.575G>A was annotated as a single nucleotide polymorphism (SNP) in the dbSNP database (rs41296057; http://www.ncbi.nlm.nih.gov/SNP/), this entry did not contain information about allele frequency or validation status. Furthermore, c.575G>A was not present in 13,006 control chromosomes deposited in the Exome Variant Server (http://evs.gs.washington.edu/EVS/) and 2,188 chromosomes deposited in the 1000genomes database (http://www.1000genomes.org/). These data suggested that c.575G>A is a very rare *PRPF4* variant. The predicted amino acid exchange affects a highly conserved arginine residue at position 192 (p.R192H) and was predicted by the PROVEAN, PolyPhen-2 and SIFT algorithms to have a deleterious effect [23]–[25]. Hence, we set out to analyze the impact of the p.R192H variant on PRPF4 protein function.

**Figure 2:**
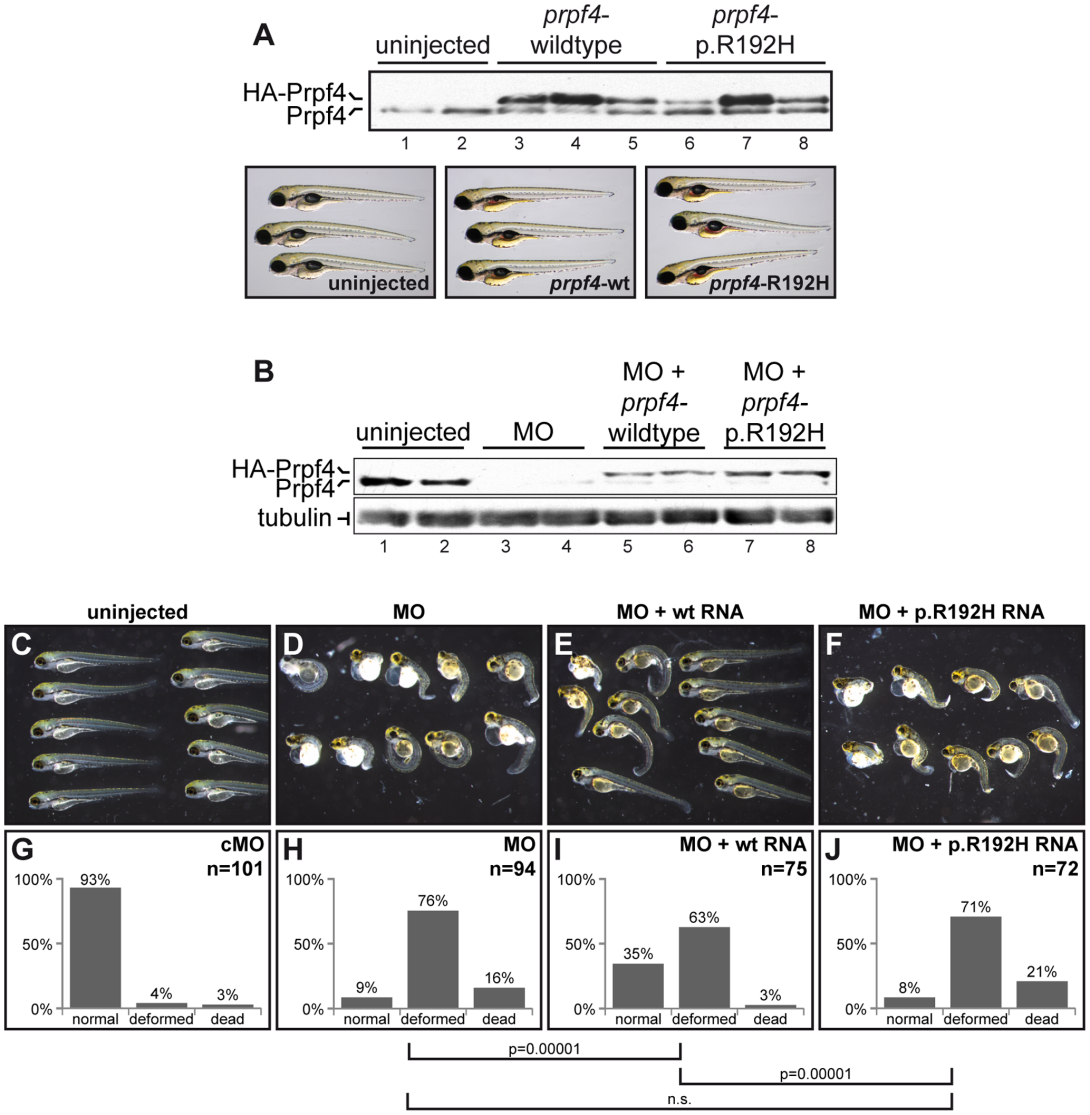
The p.R192H variant leads to a loss of function *in vivo*. (**A**) Expression of zebrafish Prpf4 carrying a mutation corresponding to p.R192H does not have a dominant negative effect. Upper panel: Western blot detection of endogenous Prpf4 and exogenous HA-tagged Prpf4 in embryos injected with RNAs indicated above. Lower panel: No defects in embryonic development were observed upon overexpression of wildtype or p.R192H Prpf4. (**B**) Western blot showing Prpf4 protein levels in zebrafish injected with the indicated combinations of *prpf4* morpholino (MO) and RNA. (**C–F**) The p.R192H mutant fails to rescue a lethal *prpf4* deficiency. Representative selections of 3 days post fertilization (dpf) larvae from embryos injected with the indicated combinations of *prpf4* morpholino (MO) and RNA. (**G–J**) Quantification of normal, deformed and dead animals from four independent rescue experiments. The rescue effect (H vs. I) and the loss of function effect of the p.R192H mutation (I vs. J) were highly significant (Pearson χ^2^ test; n is the total number of injected animals). Please note that the control experiments (uninjected, MO and MO + wt RNA), but not the characterization of p.R192H, were previously published as part of a larger knockdown study [22]. As the characterization of p.R192H has been performed in the context of this study, we reproduce here the control data for clarity (with permission from Oxford University Press).

First, we wanted to investigate whether the mutant allele acts in a dominant negative manner. For this, zebrafish embryos were injected with mRNAs transcribed *in vitro* encoding for a fusion protein of an N-terminal hemagglutinin epitope (HA-tag) and zebrafish Prpf4, either in wildtype form or with an amino acid exchange that corresponds to p.R192H. The HA-tag allowed for the simultaneous and semi-quantitative detection of exogenous and endogenous protein by Western blotting using a Prpf4-specific antibody. This revealed an overexpression of exogenous HA-prpf4 (wildtype and mutant) in fish that were injected with the corresponding mRNAs (Figure 2A, upper panel). Yet, neither of the proteins interfered with normal embryonic development (Fig.2 A, lower panel), suggesting that p.R192H does not have a strong dominant negative effect.

Next, we addressed the possibility that p.R192H results in a loss of function. For this, we included the p.R192H mutant in our rescue analysis of severe Prpf4 deficiency [22]. In this assay, zebrafish embryos were injected with a dose of antisense morpholino that strongly reduces Prpf4 expression (Fig. 2B, compare lanes 1 and 2 with lanes 3 and 4) and leads to an embryonic lethal phenotype (Fig. 2, compare C and D). Although the co-injection of *in vitro* transcribed, morpholino-insensitive mRNAs encoding HA-Prpf4 in either wildtype or mutant form led to the expression of comparable amounts of protein (Fig. 2B, compare lanes 5 and 6 with lanes 7 and 8), only the injection of wildtype mRNA resulted in an improvement of the phenotype (Fig. 2, compare E and F). Quantification of these results (Fig. 2 G–J) confirmed the significantly lower rescue effect of mutant Prpf4 compared to wildtype protein (Pearson χ^2^ test, p = 0.00001), suggesting that p.R192H results in a loss of function.

### The p.R192H variant disrupts binding of PRPF4 to PRPF3

**Figure 3:**
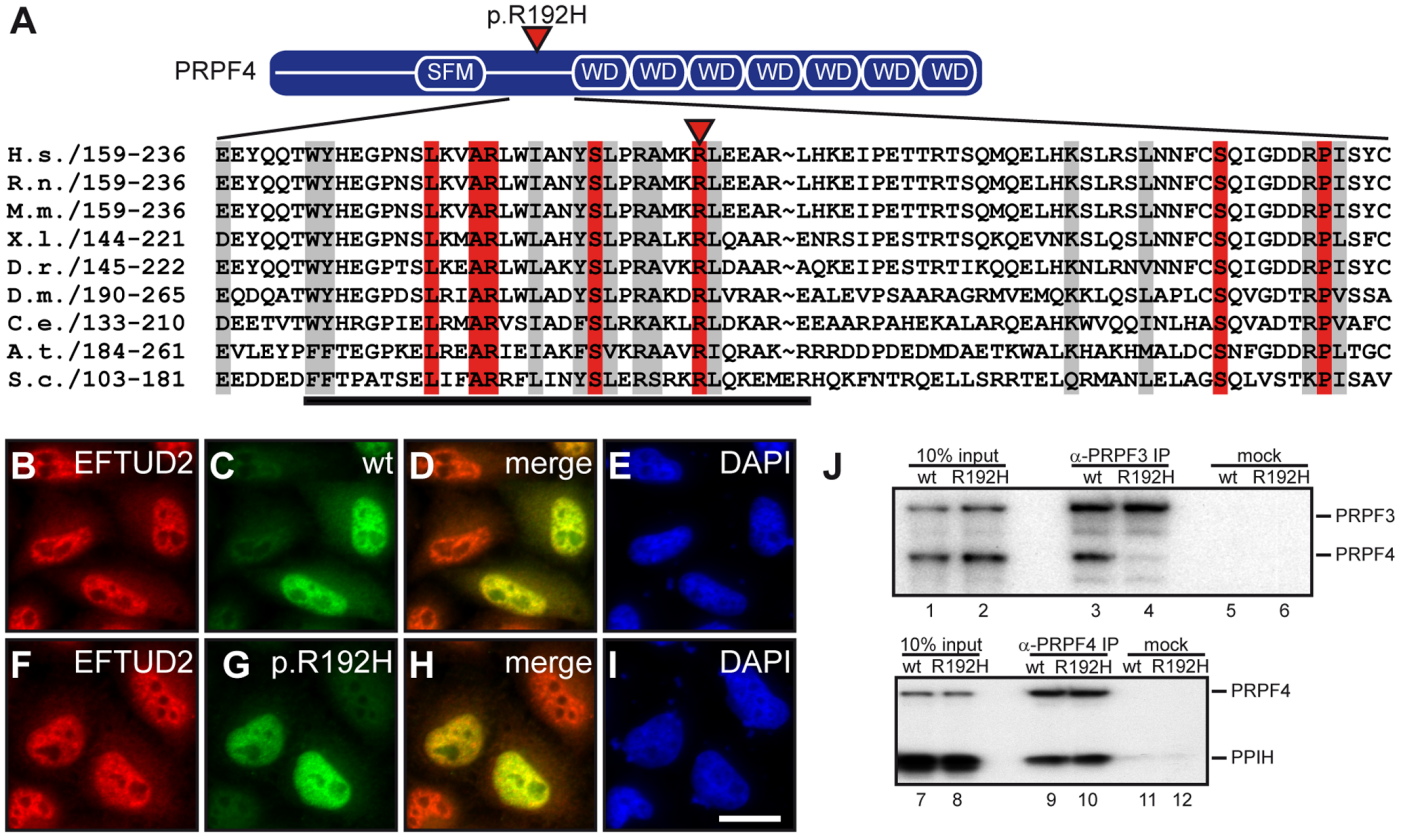
Characterization of the *PRPF4* missense variant p.R192H identified in an RP patient. (**A**) Domain organization of PRPF4 (upper panel). The p.R192H amino acid exchange is located between the splicing factor motif (SFM) and the WD40 repeat domain (WD). Amino acid sequence alignment revealed a strong conservation of the affected arginine residue across species (lower panel). Identical residues are shown in red; similarity greater than 70% (BLOSUM62) is indicated by a grey box. See table S1 for sequence accession numbers. (**B–I**) The subcellular localization of PRPF4 is not affected by the p.R192H mutant. Wildtype (B–E) and the p.R192H form of PRPF4 (F–I) were detected in the nuclei of transfected HeLa cells. Both proteins co-localized with EFTUD2 protein in a speckled pattern typical for splicing factors (B, F). Nuclei were control-stained with DAPI (E, I). The scale bar in K is 20 µm and accounts for all images. (J) The p.R192H missense mutant impairs binding to PRPF3. Immunoprecipitation analysis of *in vitro* translated [^35^S]-labeled PRPF4-PRPF3 complex (upper panel) using an anti-PRPF3 antibody revealed a significant reduction of co-precipitated p.R192H-PRPF4 (compare lanes 3 and 4). The interaction of PRPF4 to PPIH in contrast, was not affected (compare lanes 9 and 10). Lanes 1, 2, 7 and 8 show 10% of the inputs, lanes 5, 6, 11 and 12 are mock immunoprecipitations.

Phylogenetic alignment of the amino acid sequence of PRPF4 homologs revealed that arginine 192 of human PRPF4 is highly conserved in evolution (Fig. 3A) and locates in a 34 amino acids stretch essential for Prp4 function in yeast [26]. Since this basic region contains a putative nuclear localization signal [27], it was first tested whether p.R192H interferes with nuclear import and/or subnuclear localization of PRPF4. The localization of human, HA-tagged wildtype and p.R192H PRPF4 was analyzed in transfected HeLa cells by indirect immunofluorescence. Both proteins were found in the nucleus in a speckled pattern that is characteristic for splicing factors and co-localized with the U5 snRNP associated splicing factor EFTUD2 (Fig. 3B–I). These results indicate that the missense mutation p.R192H does not affect the sub-cellular localization of PRPF4.

PRPF4 is a constituent of the U4/U6 di-snRNP and as such becomes also integrated into the U4/U6.U5 tri-snRNP. In the context of these snRNPs, PRPF4 directly interacts with PRPF3 (the product of the RP disease gene *PRPF3*), and the cyclophilin PPIH [28], [29]. To analyze whether p.R192H interferes with these interactions, we generated [^35^S]-labeled, HA-tagged wildtype and mutant PRPF4 by translation in rabbit reticulocyte lysates *in vitro*, along with their potential interacting proteins. The proteins of interest were then immunoprecipitated by anti-PRPF3 antibodies and detected by autoradiography. As shown in Figure 3 J, anti-PRPF3 antibodies efficiently co-precipitated wildtype PRPF4 but not the p.R192H mutant (upper panel, compare lanes 3 and 4). The interaction of PRPF4 with PPIH was not affected, suggesting that PRPF4 p.R192H is not globally misfolded (Fig. 3J, lower panel). Thus, the p.R192H amino acid exchange results in a specific disruption of the binding of PRPF4 to PRPF3.

### The p.R192H variant leads to a snRNP integration defect of PRPF4

**Figure 4:**
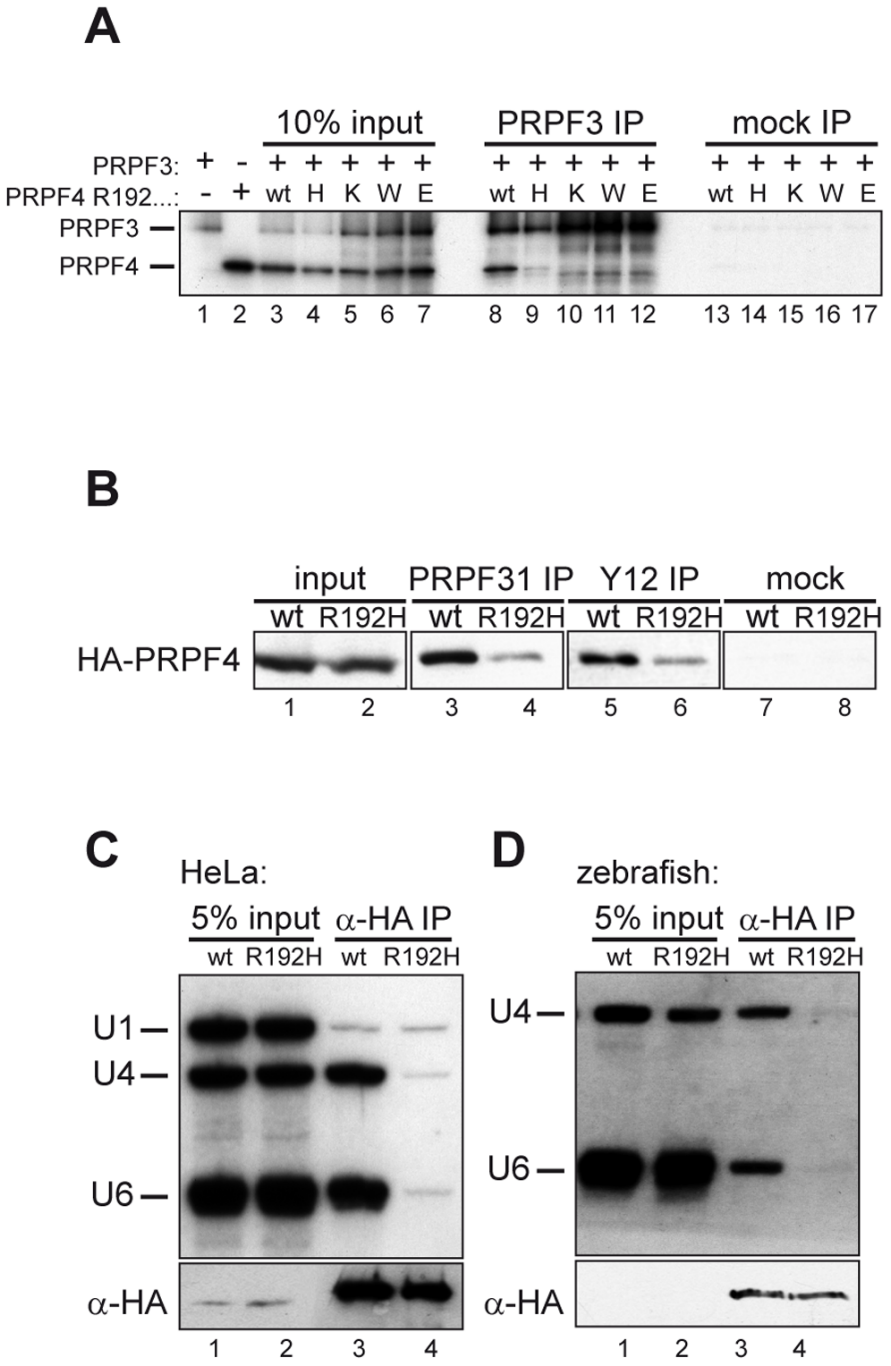
The arginine residue at position 192 is essential for integration of PRPF4 into snRNPs. (**A**) A series of mutants at position 192 was analyzed for binding to PRPF3. Neither lysine (positively charged) nor tryptophan (aromatic) nor glutamate (negatively charged) were tolerated at this position. (**B**) Reduced co-precipitation of p.R192H PRPF4 with snRNPs. HEK293 cells were transfected with wildtype and mutant HA-PRPF4 and lysates were immunoprecipitated using an anti-PRPF31 antibody (lanes 3 and 4), anti-Sm-antibody Y12 (lanes 5 and 6) or mock precipitated (lanes 7 and 8). Lanes 1 and 2 show 10% of the input used for the immunoprecipitations. (**C**) PRPF4 p.R192H fails to associate with U4 and U6 snRNA. HEK293 cells were transfected with HA-tagged PRPF4 in either wildtype or p.R192H mutant form. Anti-HA immunoprecipitation was controlled by Western blot (lower panel) and co-precipitated di-snRNP RNAs U4 and U6 were analyzed by Northern blotting (upper panel). U1 snRNA served as a specificity control. (**D**) snRNA co-immunoprecipitation experiments using zebrafish embryo lysates. Embryos were co-injected with 50 pg wildtype or p.R192H HA-*prpf4* mRNA and 0.5 ng *prpf4*-MO to facilitate snRNP integration of exogenous protein. At 10 hpf, extracts were prepared and immunoprecipitations were analyzed as described in (C).

The data presented above pointed to a crucial function of the residue 192 in the interaction of PRPF4 with PRPF3. The missense mutation identified may disrupt this interaction by local unfolding, or by preventing a defined interaction of arginine 192 with the corresponding interaction surface in PRPF3. To distinguish between both possibilities and to assess the importance of the physicochemical properties of this residue, we produced a series of mutants where arginine 192 was replaced with lysine, tryptophan or glutamate. These mutants were co-translated with PRPF3 and assayed for binding by co-immunoprecipitation with a PRPF3 antibody. Although the p.R192H mutant showed the strongest effect, a decrease in PRPF3 binding was also observed for all other variants tested (Fig. 4A). These data suggest that the arginine at position 192 engages in a specific contact with the PRPF3 binding interface that is prevented in the p.R192H variant.

Mutational studies in yeast have suggested that Prp4 is integrated into the U4/U6 snRNP via its binding to Prp3 [27]. It was hence a possibility that the p.R192H mutant of PRPF4 would not only disrupt the interaction with PRPF3 but also interfere with its integration into spliceosomal complexes. Two independent experiments were performed to test this possibility. In a first approach, HEK293 cells were transfected with HA-tagged versions of either wildtype or p.R192H PRPF4. Cellular lysates were then immunoprecipitated with antibodies directed against either PRPF31 (a U4/U6-snRNP specific component that does not directly bind to PRPF4) or the Sm proteins common to spliceosomal snRNPs (Fig. 4 B). A robust co-precipitation of wildtype protein by both antibodies was observed, whereas the p.R192H mutant PRPF4 was poorly co-precipitated (compare lanes 3 with 4 and 5 with 6, respectively; see also lanes 1 and 2 for inputs). In a second approach, wildtype and p.R192H mutant HA-PRPF4 were immunopecipitated with anti-HA antibodies from transfected HeLa cells and analyzed for co-precipitation of the tri-snRNP snRNAs U4 and U6 as well as snRNA U1 as a control by Northern blotting (Fig. 4 C). Although wildtype and mutant proteins were precipitated with similar efficiency (lower panel), co-precipitation of the snRNAs U4 and U6 was markedly reduced for mutant PRPF4 (compare lanes 3 and 4). Thus, the p.R192H PRPF4 variant is not stably incorporated into snRNPs.

We next considered the possibility that the uncovered biochemical defect might also underlie the inability of p.R192H mutant *prpf4* mRNA to rescue the lethal Prpf4 knockdown in zebrafish. This was addressed by analyzing the U snRNP integration of wild type and mutant Prpf4 in zebrafish embryos. Co-injections of morpholino against endogenous *prpf4* together with morpholino-insensitive *in vitro* transcripts encoding either wildtype or p.R192H HA-Prpf4 were performed in an analogous manner as described for the rescue experiments. At 10 hours post fertilization (hpf), lysates of the embryos were prepared and subjected to anti-HA immunoprecipitation. Co-precipitated snRNAs were then analyzed by northern blotting using probes specific for zebrafish U4 and U6 snRNAs. Similar levels of HA-Prpf4 were recovered from animals injected with wildtype or p.R192H RNA (Fig. 4D, lower panel), but the amount of associated di-snRNP snRNAs was severely reduced for p.R192H HA-Prpf4 (Fig. 4D, upper panel). Thus, the p.R192H mutation interferes with the integration of PRPF4 into spliceosomal complexes *in vivo*. Given the essential nature of this step in pre-mRNA processing, this defect is a likely cause for the inability of p.R192H RNA to rescue Prpf4 deficiency.

## Discussion

In this study, we describe the identification of an isolated RP patient that carries a heterozygous missense variant (p.R192H) in the gene encoding the tri-snRNP splicing factor PRPF4. We show that this variant disrupts the binding of PRPF4 to its interactor PRPF3 *in vitro*. This leads to an impaired integration into spliceosomal particles and a loss of physiological function *in vivo*.

PRPF4 has been shown previously to form a very stable ternary complex with PRPF3 and the peptidyl-prolyl isomerase PPIH. Within this ternary complex, PRPF4 acts as a bridging factor by contacting PPIH via its N-terminal part and PRPF3 via its central and C-terminal part [28], [29]. Consistent with this mapping is our observation that the p.R192H mutation affected PRPF3 binding but not the association with PPIH. This mode of binding suggests that p.R192H not only causes a defective integration of PRPF4 into the tri-snRNP, but that it might also lead to the concomitant loss of PPIH, a protein necessary for efficient splicing [30]. Remarkably, a similar mechanism has been proposed for an RP-mutant form of PRPF31 that is thought to sequester its interactor PRPF6 out of the tri-snRNP [31], and an altered composition of the tri-snRNP has also been described for RP-causing missense mutations in PRPF8 [21].

Our biochemical data strongly indicate that the p.R192H variant leads to the production of non-functional protein and thus represents a functional null allele. However, patient RP-106 has two daughters, one of which was found to have inherited the c.575G>A variant (Fig. 1). Both daughters underwent detailed ophthalmological investigation and did not show signs of retinal degeneration. While the genetic study presented here thus does not allow to conclude whether PRPF4 is causally linked to RP, additional evidence indicates that this is indeed the case: Our data obtained in zebrafish showed that the sub-lethal knockdown of Prpf4 leads to a retina-specific phenotype similar to that of RP [22]. The data presented here shows that wildtype Prpf4, but not a variant corresponding to p.R192H can rescue such Prpf4 deficiency (Fig. 2). Further support for a link between *PRPF4* deficiency and RP comes from a recent study where *PRPF4* mutations were found to cause adRP in a Chinese cohort [10]. Also, a potentially pathogenic missense variant of PRPF4 (p.P187A) was reported in two German siblings with RP [32]. Although this latter study failed to demonstrate a clear association between *PRPF4* and RP in North American and European populations [32], these data indicate that rare defects in PRPF4 might cause RP in a limited subset of patients.

The absence of RP symptoms in the daughter who carries the p.R192H variant of PRPF4 might be due to incomplete penetrance or largely variable disease expressivity, both being phenomena frequently observed for RP-associated mutations in splicing factors [33]–[35]. For the PRPF31-linked autosomal dominant RP, this has been explained by a modifier gene that modulates PRPF31 transcription. Low expression of the modifier increases wild-type PRPF31 levels to an extent that are sufficient to prevent the development of disease symptoms in heterozygous mutation carriers [36]. It has been proposed that the vulnerability of retinal cells to splicing factor defects arises from an unmet requirement for the production of sufficient amounts of mature retinal mRNAs [20]. Thus, defects in different splicing factors might act together to decrease the level of functional tri-snRNPs below the threshold required in retinal cells. This does not necessarily require a complete loss of the mutant protein from the tri-snRNP, as demonstrated by an RP-causing missense mutation in SNRNP200 which still allows integration of the mutant protein into the tri-snRNP but causes a decrease in splicing efficiency and fidelity [37]. It is possible that a hypomorphic variant in another splicing factor is present in the patient, but not in her daughter, and leads to the manifestation of RP. Moreover, it can not be excluded that the RP of the patient is caused by biallelic mutations in a recessive RP gene, and that p.R192H represents a rare non-pathogenic variant.

Support for the notion that a decrease in overall tri-snRNP activity rather than individual splicing factor defects lead to photoreceptor damage comes from a zebrafish mutant of the tri-snRNP recycling factor SART3: Although SART3 is an assembly factor that is not itself part of the tri-snRNP [38], its mutation leads to the reduced expression of photoreceptor mRNAs [39]. Taken together, our data are consistent with a model in which rare mutations of tri-snRNP splicing factors act together to decrease the level of functional tri-snRNPs below the threshold required in retinal cells.

## Materials and Methods

### Plasmids and antibodies

Zebrafish *prpf4* was amplified from 14 hpf embryonic oligo-dT cDNA using gene-specific primers (see table S1 for sequences). Human *PRPF4* was amplified using the “Marathon human fetal brain” cDNA library (Clontech) as a template and gene-specific primers. cDNAs were inserted into a modified version of the pcDNA3 vector that adds an N-terminal hemagglutinin- (HA-) tag for the transfection of HeLa cells, into the pET21a, pET28a and the pGEX-6P1 vector for *in vitro* translation and the production of recombinant proteins and into an HA-tagged version of the pCS2+ vector for *in vitro* transcription. To generate the morpholino-insensitive *prpf4*, synonymous mutations were introduced in the ATG region by site directed mutagenesis, resulting in 6 mismatches to the antisense morpholino sequence (see table S1 for sequences of mutagenic primers). The same approach was applied to introduce the p.R177H mutation that corresponds to the human p.R192H exchange. Antibodies against PRPF3, PRPF4 and PRPF31 were described previously [22]. Antibodies against EFTUD2 were a gift from R. Lührmann. The anti-HA antibody (MMS-101R) was purchased from HiSS Diagnostics.

### Microinjection and Western blot analysis of zebrafish embryos

Microinjection of zebrafish embryos and Western blot analysis of embryo lysates was performed as described previously [22]. Briefly, one- or two-cell stage embryos were injected with a morpholino oligo directed against the start codon region of *prpf4* (*prpf4*-MO: TGGAGCTTCATCTTCATCTGACATC; Gene Tools, LLC). Lysates for Western blots were prepared by homogenization of single larvae in 20 µl of a 1∶1 mixture of Laemmli sample buffer and buffer B (100 mM NaH_2_PO_4_, 10 mM Tris pH 8.0, 8 M Urea).

### Rescue experiments

Single clutches of embryos were divided into four groups that (1) were left uninjected, (2) were injected with a lethal dose of *prpf4*-MO (2 µg/µl), (3) were co-injected with *prpf4*-MO and wildtype *prpf4* mRNA or (4) were co-injected with *prpf4*-MO and p.R192H mutant *prpf4* mRNA. The co-injected mRNAs were transcribed *in vitro*, capped and purified as described previously [22]. Successful injection (knockdown of endogenous Prpf4 and expression of exogenous HA-Prpf4) was monitored by Western blotting at 12 hpf. After 4 dpf, larvae were scored as described [22] as either lethal, severely deformed or slightly deformed/not affected. At least three independent experiments were performed for each analysis.

### Immunocytochemistry

HeLa cells were grown on coverslips in DMEM (PAA) containing 10% fetal calf serum (PAA) at 37°C and 5% CO_2_. HA-PRPF4 inserted into the pcDNA3 vector was transfected using Nanofectin (PAA) and two days after transfection cells were fixed and immunocytochemistry was performed as described previously [40].

### *In vitro* translation, co-immunoprecipitations and Northern blotting

Proteins were translated from pET21a and pET28a vectors using the T7 TNT coupled transcription and translation kit (Promega) in the presence of [^35^S]-L-methionine.

Extracts form HEK293 cells were obtained by sonication in IPP300 (20 mM Tris pH 7.0, 300 mM NaCl, 0.05% NP40) containing 1.5 mM MgCl_2_, 0.5 mM DTT and protease inhibitors. Zebrafish embryo lysates were prepared from ∼100 embryos (10hpf) by sonication in 200 µl of IPP300 containing 0.5% NP40. Crude lysates were extracted using 100 µl N-heptane and centrifuged at 16.000 g and 4°C for 30 minutes.

For immunoprecipitations, antibodies were coupled to either protein A- or protein G-Sepharose and incubated with translated proteins/cellular extract in IPP300. After excessive washing with IPP300, bound proteins were eluted using Laemmli sample buffer and analyzed by Western blotting or autoradiography.

For Northern blot analyses, PK-buffer (2x: 200 mM Tris-HCl pH 7.5, 25 mM EDTA, 300 mM NaCl, 2% SDS) and phenol were added to the immunoprecipitates. After phase separation, the RNA was ethanol-precipitated, subjected to PAGE (10%, 8 M Urea) and electroblotted onto Hybond N+ Nylon membrane (GE Healthcare). Hybridization of [^32^P]-labeled oligonucleotides specific for U1, U4 and U6 snRNAs (see table S1 for sequences) was carried out at 42°C in RapidHyb solution (GE Healthcare).

### Patient screening and analysis

Samples were obtained with written informed consent. Clinical investigations were conducted according to the Declaration of Helsinki, and the study was approved by the institutional review board of the Ethics Committee of the University Hospital of Cologne. 85 patients with sporadic or autosomal dominant RP were screened for mutations in the *PRPF4* gene. All 14 coding exons were PCR-amplified and the products were subjected to SSCP (single strand conformation polymorphism) analysis. Products with band shifts were directly sequenced. All 85 patients with no previously identified RP-mutations and 100 healthy control individuals were genotyped for the presence of p.R192H by direct sequencing.

## Supporting Information

Table S1 **Oligonucleotide sequences and accession numbers used.**
(PDF)
