# Supplementary material for: Identification of a PRPF4 Loss-of-Function Variant That Abrogates U4/U6.U5 Tri-snRNP Integration and Is Associated with Retinitis Pigmentosa
Source: PLoS One. 2014 Nov 10;9(11):e111754. doi: 10.1371/journal.pone.0111754 (PMC4226509; doi:10.1371/journal.pone.0111754)
Supplement: Table S1 — Oligonucleotide sequences and accession numbers used. (PDF) [file pone.0111754.s001.pdf]

Antisense morpholino oligos:

prpf4 -MO:	5'-TGGAGCTTCATCTTCATCTGACATC-3'
control-MO:	5'-CCTCTTACCTCAGTTACAATTTATA-3'

Oligos used for northern blot hybridization:

U1:	5'-ATCATGGTATCTCCCCTGCCAGGTAAGTAT-3'
U4:	5'-GGCGGGGTATTGGGAAAAGTTTTCAATTAGC-3'
U6:	5'-GCTAATCTTCTCTGTATCGATCCAATTTTAGTA-3'

Oligos used for cloning of zebrafish *prpf4* and human *PRPF4* :

prpf4 fw:	5'-AATAGAATTCATGTCAGATGAAGATGAAGCTCC-3'
prpf4 rev:	5'-AATACTCGAGTTCTGACATCCAGAGTTTAAAGG-3'
PRPF4 fw:	5'-AATACGGATCCATGGCTTCCTCGCGAGCCTC-3'
PRPF4 rev:	5'-AATAACTCGAGTTCAGCCATCCACAGCTTGAAG-3'

Oligos used for *prpf4* morpholino binding site mutagenesis

HA *prpf4* M1-InsA-SubsS2 fw	5'-CTAGCTTGGGATCCGAATTCATGGCTAGTGATGAAGATGAAGCTCCAGTTG-3'
HA *prpf4* M1-InsA-SubsS2 rev	5'-CAACTGGAGCTTCATCTTCATCACTAGCCATGAATTCGGATCCCAAGCTAG-3'

Accession numbers for the PRPF4 alignment:

Homo sapiens	GI48146327
Rattus norvegicus	GI34868449
Mus musculus	GI55925589
Xenopus laevis	GI51703478
Danio rerio	GI41054303
Drosophila melanogaster	GI7293972
Caenorhabditis elegans	GI17505895
Saccharomyces cerevisiae	GI6325435
